# Supplementary material for: Associations between resting state functional brain connectivity and childhood anhedonia: A reproduction and replication study
Source: PLoS One. 2023 May 4;18(5):e0277158. doi: 10.1371/journal.pone.0277158 (PMC10159190; doi:10.1371/journal.pone.0277158)
Supplement: S1 Fig — Pearson correlations for student’s t-statistics (left) and lnBF statistics (right) derived from the previous author’s analyses and those derived from our reproduction analyses for A) anhedonia, B) depressed mood, and C) anxiety. LnBF are natural logarithm of Bayes Factors. (DOCX) [file pone.0277158.s001.docx]

**Supplementary Figure. 1 - Correlations between statistics from the current analyses and those reported by the previous authors.** Pearson correlations for student’s t-statistics (left) and lnBF statistics (right) derived from the previous author’s analyses and those derived from our reproduction analyses for **A)** anhedonia, **B)** depressed mood, and **C)** anxiety. LnBF are natural logarithm of Bayes Factors.

**
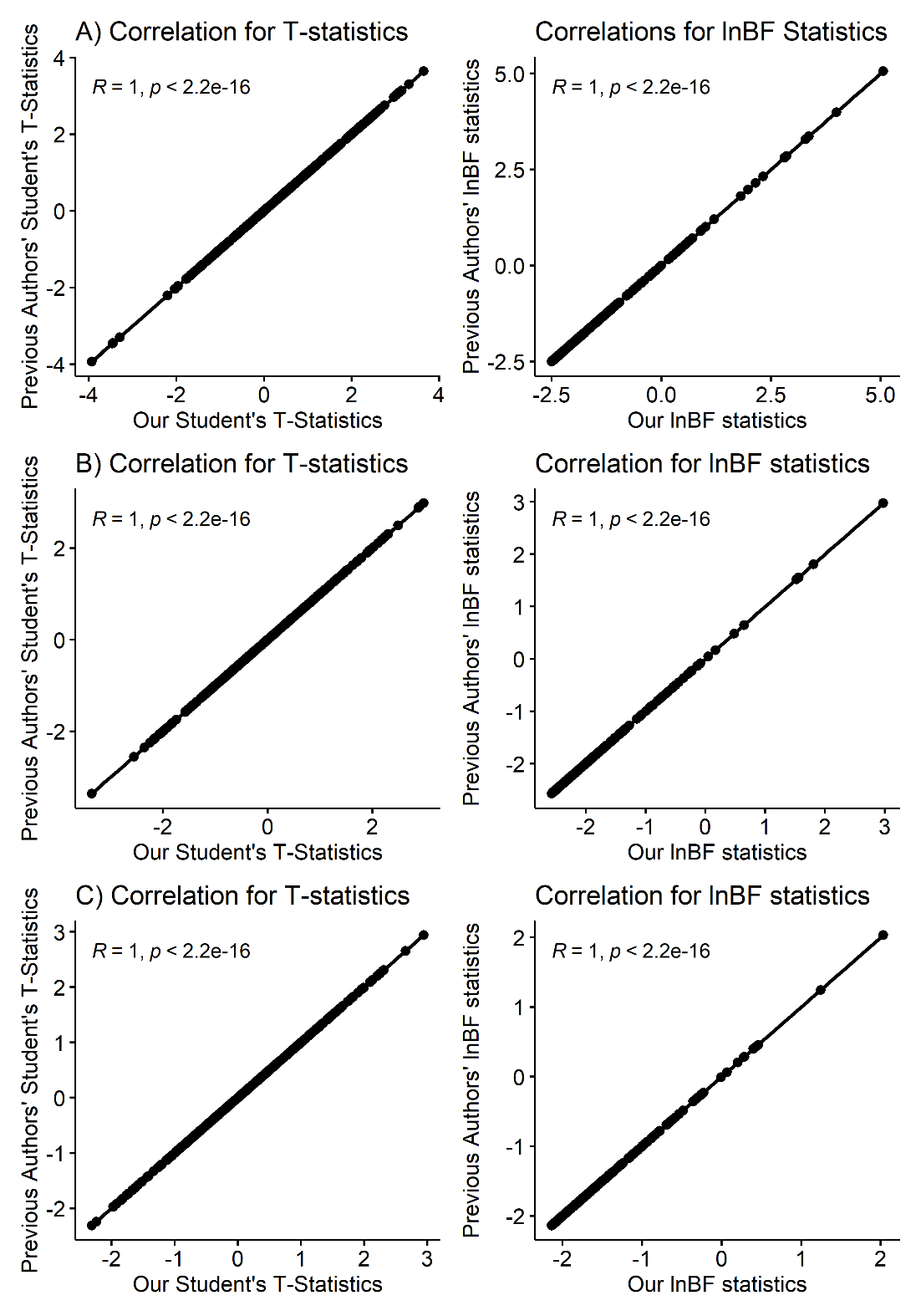
**
