# Supplementary material for: Associations between resting state functional brain connectivity and childhood anhedonia: A reproduction and replication study
Source: PLoS One. 2023 May 4;18(5):e0277158. doi: 10.1371/journal.pone.0277158 (PMC10159190; doi:10.1371/journal.pone.0277158)

**Supplementary Figure. 3 – Comparing Welch’s t-tests to Student’s t-tests.** To assess the potential bias introduced by unequal variances across anhedonia and control groups, we performed Welch’s t-tests for those rsfMRI measures with significant F-tests in the **A)** ABCD 1.0, **B)** ABCD 4.0 (excluding 1.0), and **C)** full ABCD 4.0 samples and then correlated the Welch’s t-statistics with Student’s t-statistics (left) and the Welch’s p-values with Student’s p-values (right).


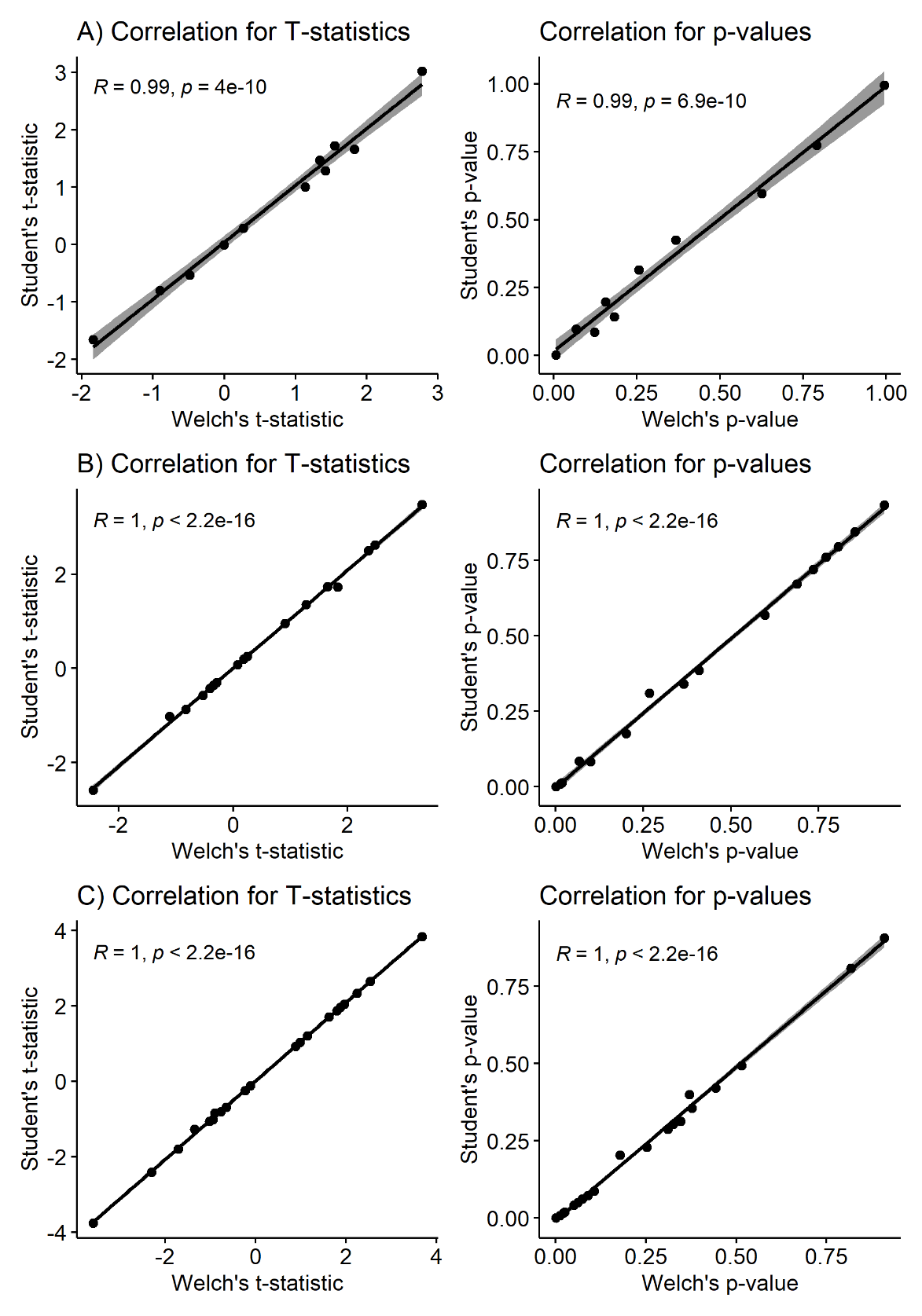

Supplement: S3 Fig — To assess the potential bias introduced by unequal variances across anhedonia and control groups, we performed Welch’s t-tests for those rsfMRI measures with significant F-tests in the A) ABCD 1.0, B) ABCD 4.0 (excluding 1.0), and C) full ABCD 4.0 samples and then correlated the Welch’s t-statistics with Student’s t-statistics (left) and the Welch’s p-values with Student’s p-values (right). (DOCX) [file pone.0277158.s003.docx]
