# Supplementary material for: Associations between resting state functional brain connectivity and childhood anhedonia: A reproduction and replication study
Source: PLoS One. 2023 May 4;18(5):e0277158. doi: 10.1371/journal.pone.0277158 (PMC10159190; doi:10.1371/journal.pone.0277158)
Supplement: S6 Fig — Here, we visualize the residuals plotted against marginal fitted values of the linear regression model for the FrontoParietalRightVentraldc rsfMRI measure using the ABCD 1.0 sample (left), which exhibited a significant BP test for heteroskedasticity. We also performed weighted-least-squares regression (WLS) and correlated the t-statistics for each model predictor with those from the original ordinary-least-squares (OLS) regression (right). (DOCX) [file pone.0277158.s006.docx]

**Supplementary Figure. 6 – Visual inspection of residuals from the regression (controlling for sociodemographic covariates) for the *FrontoParietalRightVentraldc* rsfMRI connectivity measure using the ABCD 1.0 and correlations between OLS and WLS t-statistics**. Here, we visualize the residuals plotted against marginal fitted values of the linear regression model for the *FrontoParietalRightVentraldc* rsfMRI measure using the ABCD 1.0 sample (left), which exhibited a significant BP test for heteroskedasticity. We also performed weighted-least-squares regression (WLS) and correlated the t-statistics for each model predictor with those from the original ordinary-least-squares (OLS) regression (right).

**
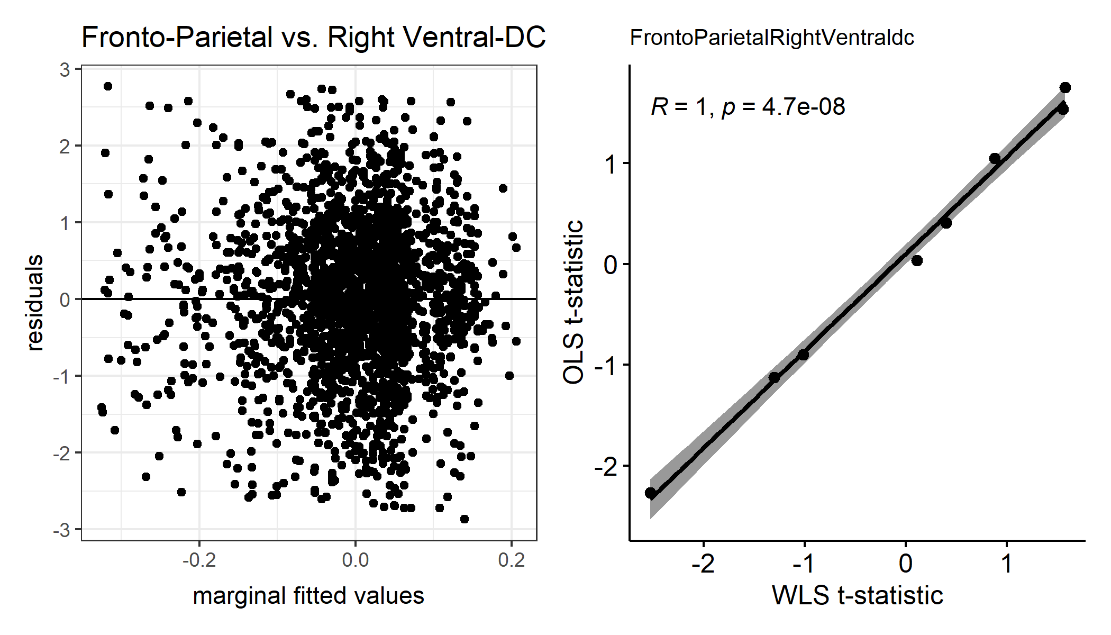
**
