# Supplementary material for: Associations between resting state functional brain connectivity and childhood anhedonia: A reproduction and replication study
Source: PLoS One. 2023 May 4;18(5):e0277158. doi: 10.1371/journal.pone.0277158 (PMC10159190; doi:10.1371/journal.pone.0277158)

**Supplementary Figure. 7 – Visual inspection of residuals from the regression for *FrontoParietalRightVentraldc* rsfMRI measure (controlling for sociodemographic and psychiatric comorbidities) using the ABCD 1.0 sample and correlations between OLS and WLS t-statistics**. Here, we visualize the residuals plotted against marginal fitted values of the linear regression model for the *FrontoParietalRightVentraldc* rsfMRI measure using the ABCD 1.0 sample (left), which exhibited a significant BP test for heteroskedasticity. We also performed weighted-least-squares regression (WLS) and correlated the t-statistics for each of the model predictors with those from the original ordinary-least-squares (OLS) regressions (right).


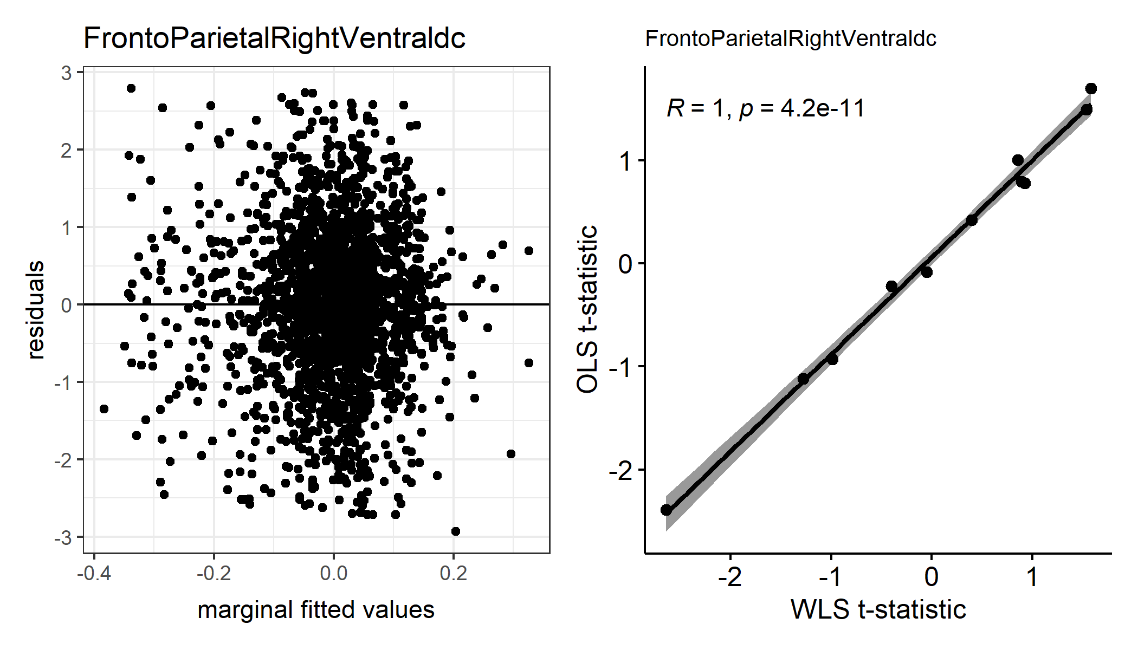

Supplement: S7 Fig — Here, we visualize the residuals plotted against marginal fitted values of the linear regression model for the FrontoParietalRightVentraldc rsfMRI measure using the ABCD 1.0 sample (left), which exhibited a significant BP test for heteroskedasticity. We also performed weighted-least-squares regression (WLS) and correlated the t-statistics for each of the model predictors with those from the original ordinary-least-squares (OLS) regressions (right). (DOCX) [file pone.0277158.s007.docx]
