# Supplementary material for: Associations between resting state functional brain connectivity and childhood anhedonia: A reproduction and replication study
Source: PLoS One. 2023 May 4;18(5):e0277158. doi: 10.1371/journal.pone.0277158 (PMC10159190; doi:10.1371/journal.pone.0277158)

**Supplementary Figure. 11 – Visual inspection of residual variance from regressions (controlling for sociodemographic covariates and psychiatric comorbidities) using the full ABCD 4.0 sample for rsfMRI measures with significant Breusch-Pagan tests and correlations between OLS and WLS t-statistics**. Here, we visualize the residuals plotted against marginal fitted values for 4 rsfMRI connectivity measures exhibiting significant BP tests for heteroskedasticity. We also performed weighted-least-squares regression (WLS) for each rsfMRI measure and correlated the t-statistics for the model predictors with those from the original ordinary-least-squares (OLS) regressions.


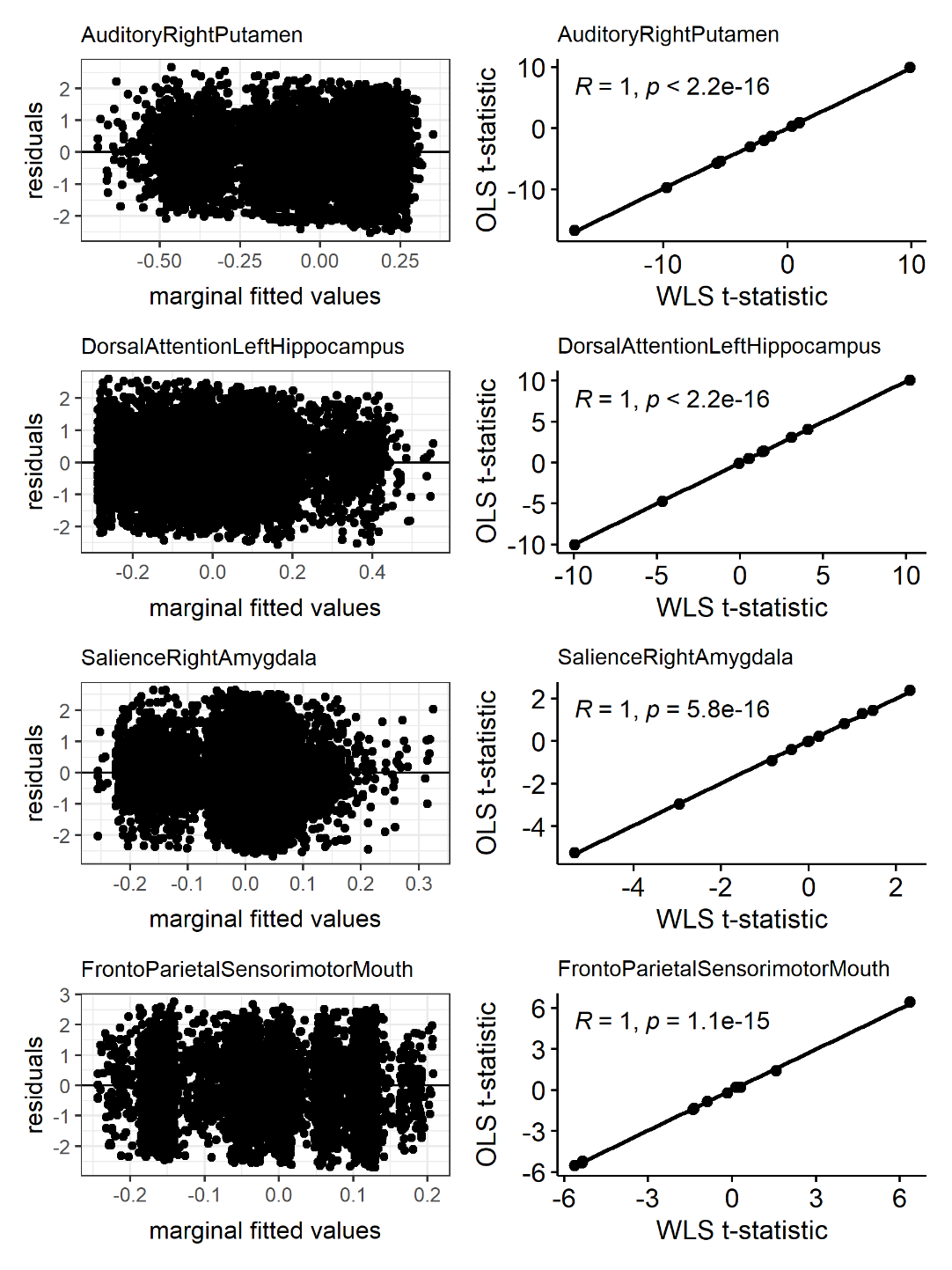

Supplement: S11 Fig — Here, we visualize the residuals plotted against marginal fitted values for 4 rsfMRI connectivity measures exhibiting significant BP tests for heteroskedasticity. We also performed weighted-least-squares regression (WLS) for each rsfMRI measure and correlated the t-statistics for the model predictors with those from the original ordinary-least-squares (OLS) regressions. (DOCX) [file pone.0277158.s011.docx]
