# Supplementary material for: Associations between resting state functional brain connectivity and childhood anhedonia: A reproduction and replication study
Source: PLoS One. 2023 May 4;18(5):e0277158. doi: 10.1371/journal.pone.0277158 (PMC10159190; doi:10.1371/journal.pone.0277158)

**Supplementary Figure. 15 – Visual inspection of normality for rsfMRI connectivity measure residuals from multiple linear regression models with sociodemographic covariates and psychiatric comorbidities using the ABCD 4.0 (excluding 1.0) sample.** Density (left) and quantile-quantile (right) plots are shown for 3 representative rsfMRI connectivity measures with significant associations with anhedonia.


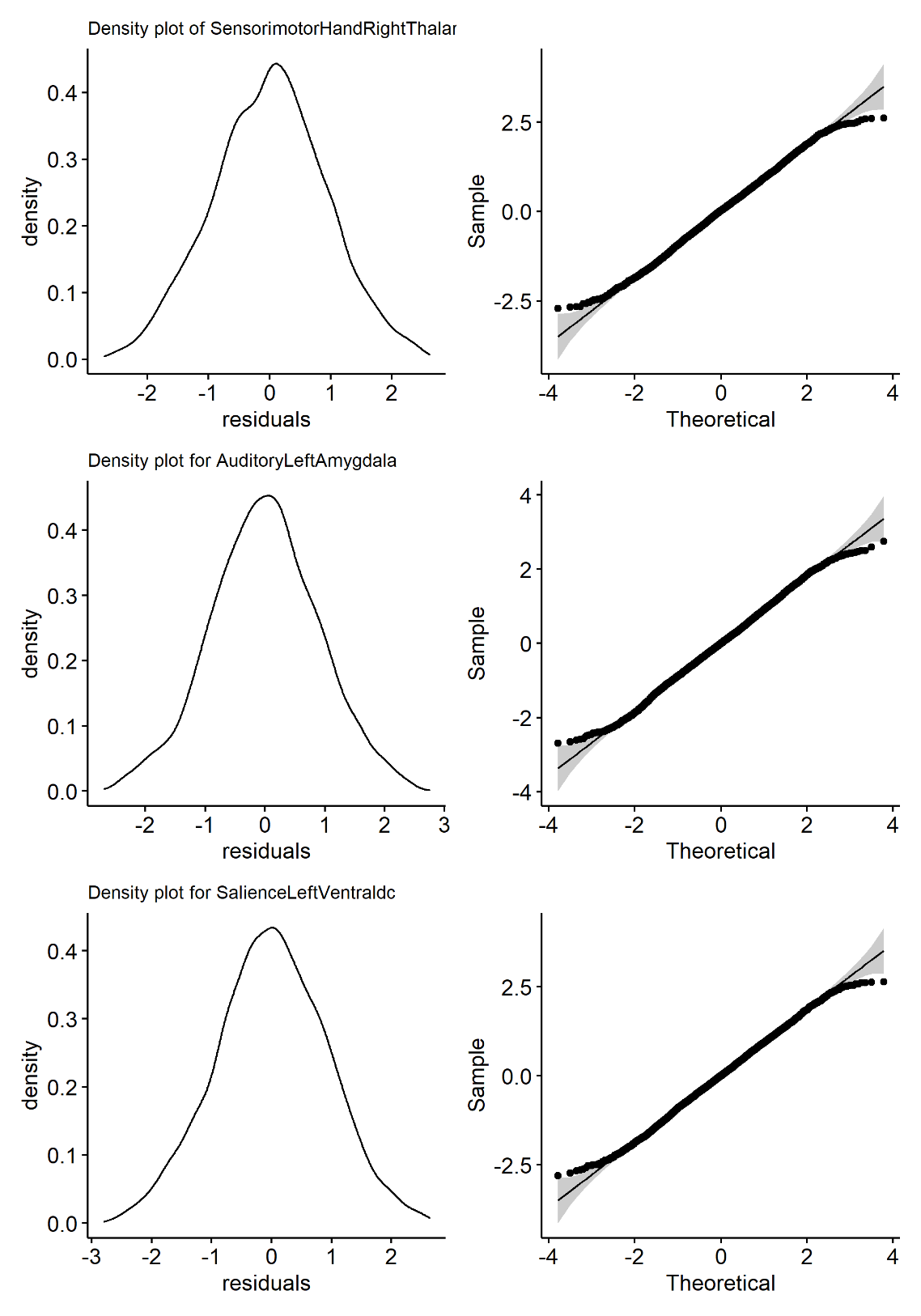

Supplement: S15 Fig — Density (left) and quantile-quantile (right) plots are shown for 3 representative rsfMRI connectivity measures with significant associations with anhedonia. (DOCX) [file pone.0277158.s015.docx]
